# Excellence and equity in eye care

**Published:** 2009-03

**Authors:** Nick Astbury

**Affiliations:** Consultant Ophthalmic Surgeon, Norfolk and Norwich University Hospital NHS Trust, Colney Lane, Norwich NR4 7UY, UK.

**Figure:**
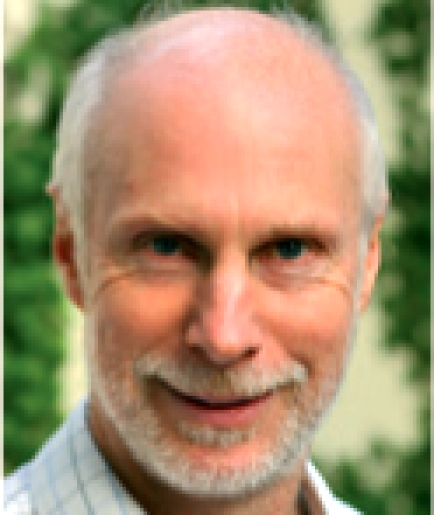


The theme of the 8^th^ General Assembly was ‘Excellence and equity in eye care’ -superlative sound bites perhaps, but when those words are considered in the context of VISION 2020, they take on a meaning that should define all our efforts to combat avoidable blindness.

## Striving for excellence

Allen Foster, in his inspiring ‘Sir John Wilson’ oration, urged that excellence should mean more than simply quality in clinical care or the latest technology.

Excellence, he said, should involve aspiring for the ideal in research, management, evidence-based clinical practice, and non-clinical patient care. It should be both our individual and corporate goal. Everyone involved in eye care should not compromise their own high standards in striving for excellence in their work.

Of course, there are many barriers to overcome, not least the lack of infrastructure and resources that besets so many developing countries. But that is no reason not to strive for excellence and respect the basic human right of all individuals to receive high-quality eye care.

## VISION 2020 is as important as ever

GN Rao, in his address to the Assembly, defined excellence in terms of concept planning, infrastructure, quality of human resources, and operating systems - in fact, all the building blocks of VISION 2020 that require the use of ‘heart, head and hands’.

He wrote in 2005^1^: “70% of the population [in developing countries] live in rural areas, about half of whom are economically deprived with significant social barriers. There are very poor public education or information systems and funding for blindness programmes in most of these countries is virtually non-existent.”

There has been little improvement since and, in 2009, the need for VISION 2020: The Right to Sight remains as acute as ever.

## Equity and moral justice

Equity is about the fair distribution of resources throughout a group of people according to population, not individual, need. Equity means not discriminating between people of different ethnicity, religion, age, gender, or social class, and delivering services in an acceptable, accessible, and affordable way. Equity, Foster reiterated, is a right founded on moral justice.

**Figure:**
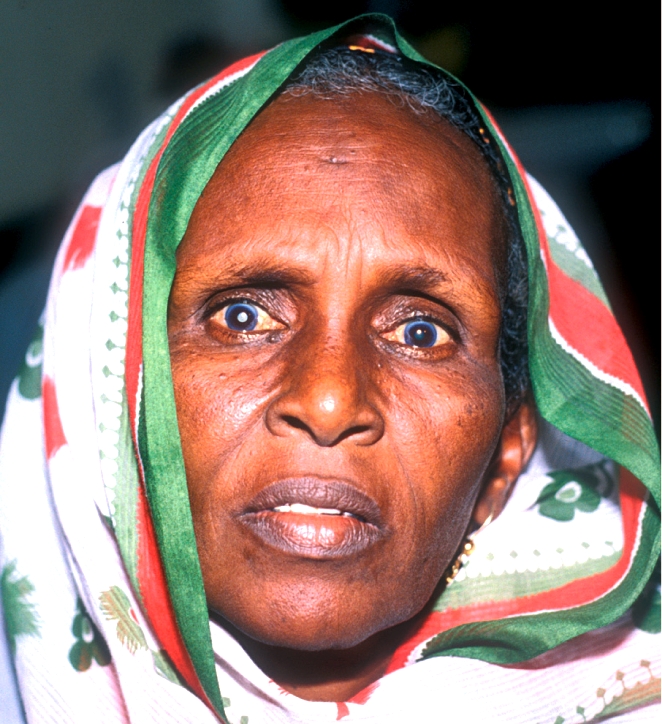
Worldwide, visual outcomes of cataract surgery are still a cause for concern. SOMALIA

Murray McGavin

## Challenges to service delivery

Two recent papers illustrate the challenges that we still face in the delivery of an excellent and equitable service.

A review by Hans Limburg et al. of recent surveys on blindness and visual impairment in Latin America^2^ analysed data from nine countries and concluded that 43% to 88% of all blindness in Latin America is curable, being caused by cataract and refractive errors. Although simple and cost-effective strategies do exist, they need to be made available to more people. In addition, the visual outcomes of cataract surgery in most of the survey areas gave cause for concern, particularly in the case of cataract surgery without intraocular lens implantation.

The Pakistan national blindness and visual impairment survey^3^ revealed a prevalence of total blindness more than three times higher in poor clusters than in affluent clusters. It also showed that, in poor communities, the cataract coverage and uptake of spectacle provision were less than half compared with rich communities. Inequity of access is an important factor even for relatively straightforward interventions such as the provision of spectacles.

## Conclusion

The ideals of excellence and equity are no less relevant in developing countries than they are in Europe and the West. All patients are equally deserving of the same high standard of care.
